# Postoperative mortality after a hip fracture over a 15-year period in Denmark: a national register study

**DOI:** 10.1080/17453674.2019.1680485

**Published:** 2019-10-22

**Authors:** Ossian Gundel, Lau Caspar Thygesen, Ismail Gögenur, Sarah Ekeloef

**Affiliations:** a Center for Surgical Science, Department of Surgery, Zealand University Hospital, Koege, Denmark;; b National Institute of Public Health, University of Southern Denmark, Copenhagen K, Denmark

## Abstract

Background and purpose — In Denmark, 44 per 10,000 persons over the age of 50 years suffered a hip fracture (HF) in 2011. We characterized the patients and identified risk factors associated with 30-day, 90-day, and 1-year postoperative mortality in Denmark from 2000 to 2014.

Patients and methods — The study builds upon data from the Danish National Patients Register and the National Causes of Death Register including all acute hospitalized HF patients aged 18 years and above. Outcomes were 30-day, 90-day, and 1-year postoperative mortality. Mortality risk was analyzed with a univariable and multivariable Cox regression including predefined variables.

Results — 113,721 acute hospitalized HF patients were admitted to Danish hospitals between 2000 and 2014. The 30-day mortality risk was 9.6%, 16% at 90 days, and 27% at 1 year after HF surgery. Mortality risk was similar from 2000 to 2014 while the median lengths of stay declined from 14 (IQR 8–25) to 8 (IQR 5–11) days. Male sex, increasing age, higher Charlson Comorbidity Index, per- and subtrochanteric fracture, and operation type other than total hip arthroplasty were independently associated with postoperative mortality.

Interpretation — Short- and long-term mortality was high after hip fracture surgery and did not decline in Denmark from 2000 to 2014.

In the Danish population, 44 per 10,000 persons over the age of 50 years suffered a hip fracture (HF) in 2011 (Driessen et al. 2016). The majority of patients with HF are elderly women with multiple comorbidities including cardiovascular, pulmonary, and genitourinary diseases, diabetes, and dementia. Compared with the general population, patients with HF have an up to 3-fold increased mortality risk (Goldacre et al. 2002). Previous studies have found a 30-day mortality risk between 5.6% and 11% (Roberts and Goldacre 2003, Roche et al. 2005), and 1-year mortality risk between 22% and 33% (Goldacre et al. 2002, Brauer et al. 2009). Age and preoperative comorbidities are known to correlate with both postoperative complications and mortality (Roche et al. 2005, Sathiyakumar et al. 2016). A study from Canada showed a high and increased incidence of HF until the beginning of the 21st century (Cheng et al. 2011). Thereafter, the prevalence of HFs has increased but the incidence rate has dropped (Rosengren et al. 2017). It is uncertain whether this drop has been accompanied by a concomitant decrease in postoperative mortality. Combined with the high economic impact of HF patients (Leal et al. 2016), the incidence rate and postoperative mortality have a large impact on the national healthcare system.

We estimated the 30-day, 90-day, and 1-year postoperative mortality, identified risk factors for mortality after hip fracture, and describe the time trend in mortality risk after HF surgery in Denmark from 2000 to 2014.  

## Patients and methods

### Data sources

This cohort study used data from the Danish National Patients Register (DNPR) and the National Causes of Death Register (NCDR). In Denmark, all contacts between a citizen and the healthcare system are recorded by a unique civil registration number and stored in the national registers. In the DNPR all hospital data are registered: admission, discharge, treatment, and operation date and time, as well as diagnoses and types of examination, treatment, and operation. Since 1994, all contacts have been registered and coded using International Classification of Diseases and Related Health Problems (ICD-10) from the World Health Organization; before that ICD-8 was used (Lamberts et al. 2014). Procedures and operations are classified using the Nordic Classification of Surgical Procedures, which is developed and maintained by the Nordic Medico-Statistical Committee (NOMESCO) (Schmidt et al. 2015). In the NCDR, time of death and primary and secondary causes of death are registered. The reporting of the study follows the STROBE statement.

### Population and definitions

The population consisted of patients aged 18 or above admitted acutely with an HF to a Danish hospital between 2000 and 2014. HF was defined by the ICD-10 codes: S72.0 (collum femoris fracture), S72.1 (pertrochanteric femur fracture), S72.1A (intertrochanteric femur fracture), S72.1B (trochanteric femur fracture), S72.2 (subtrochanteric femur fracture), and S72.8A (femoral head fracture). Patients were only included in the study if they had undergone a surgical procedure for their HF with a NOMESCO operation code: KNFB (primary inserting of hip joint prosthesis), KNFC (secondary inserting of hip joint prosthesis), KNFE (operation on hip joint capsula and ligaments), KNFF (operation on hip joint synovia and joint surface), KNFG (hip joint resection, arthroplasty, and arthrodesis), KNFJ (hip fracture operation, including internal and external fixation), KNFK (bone operation on thighbone, including resection and osteosynthesis), KNFT (operation on hip joint and thighbone, including pseudarthrosis, and bone deformity), and KNFQ (amputation of hip and thighbone). Only patients undergoing HF surgery for the first time were included in the study.

Diagnoses registered within 5 years of admission were used to create an individual comorbidity score using the Charlson Comorbidity Index (CCI). The ICD-10 codes in DNPR used to construct CCI in this study have previously been found to have a high positive predictive value (Thygesen et al. 2011). The comorbidity hazard ratio was found by comparing with non-comorbid patients.

### Outcome

The outcomes were all-cause 30-day, 90-day, and 1-year mortality after surgery for an HF. As a descriptive measure, cause-specific mortality was also reported. Length of stay was recorded. 

### Statistics

Descriptive statistics of continuous variables were expressed as mean (SD) or median (interquartile range [IQR]), while categorical data were expressed as numbers and percentages. Univariable and multivariable Cox regressions were performed. We adjusted for the predefined variables: sex, age, fracture type, operation type, CCI score, and comorbidities (previous acute myocardial infarct [AMI], heart failure, cerebrovascular disease, peripheral vascular disease, chronic obstructive pulmonary disease [COPD], kidney disease, liver disease, diabetes, rheumatic disease, cancer, cancer metastases, and dementia). The proportional hazards assumption was checked graphically with the empirical score process (Lin et al. 1993). Interactions between sex and age, as well as operation type and fracture type, were analyzed. Only the interaction between sex and age was statistically significant and therefore included in the analysis. All variables were included simultaneously in the multivariable analyses.

Results were expressed as hazard ratios (HR) (95% CI). Trends over time in mortality were analyzed with logistic regression and number of HF and length of hospital stay were analyzed with ANOVA models. The assumptions of Gaussian distributed residuals were evaluated graphically; length of stay had to be log-transformed to fulfil assumptions.

Statistical analyses were performed with SAS version 9.4 (SAS Institute, Cary, NC, USA). A 2-sided p-value < 0.05 was considered statistically significant.

### Ethics, funding, and potential conflicts of interest

The study was approved by the Danish Data Protection Agency (Approval number: REG-161-2015. Case number 15-000241). In Denmark—by law—register studies without the use of biological material do not require ethics committee approval. The study has not received any funding. None of the authors has any conflicts of interest to declare. 

## Results

### Baseline data

**Figure 1.**
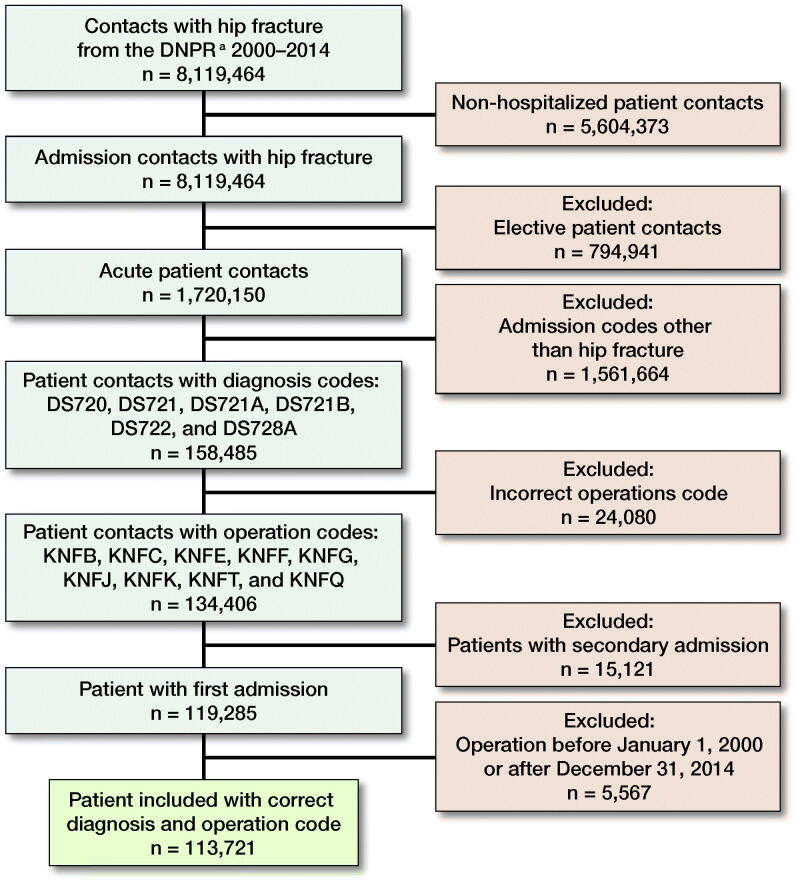
Inclusion flowchart. **^a^**Danish National Patient Register

**Figure 2.**
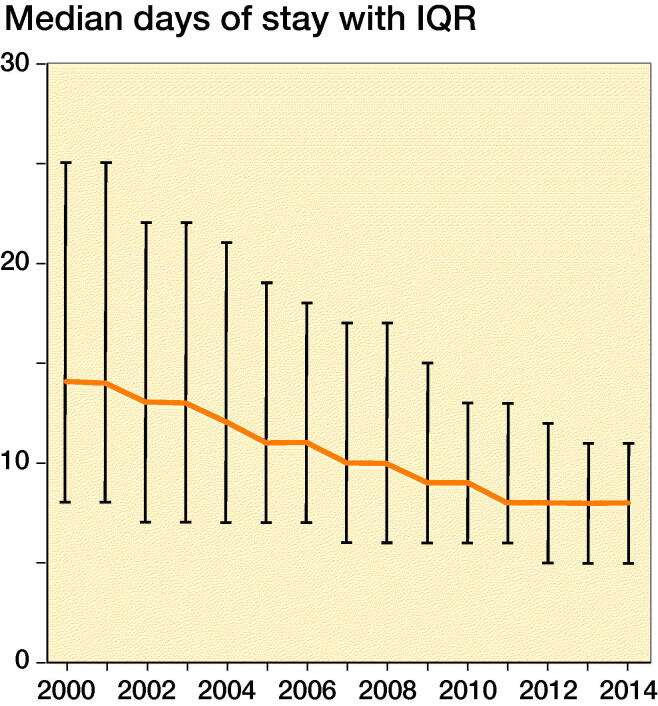
Median length of stay in days with interquartile range (IQR) after hip fracture surgery. ANOVA test of periodic effect, p < 0.01

**Figure 3.**
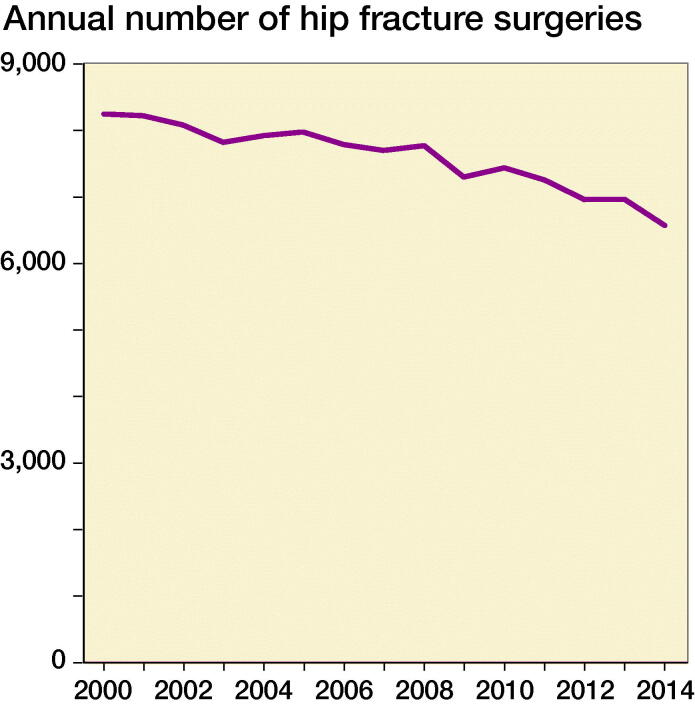
Trend in number of hip fracture surgeries from 2000 to 2014. ANOVA test of periodic effect, p < 0.001.

After excluding non-acute admissions, non-hip fracture diagnosis and operation codes, we ended up with 113,957 unique HF patients in the period 2000–2014 (Figure 1). Baseline characteristics are shown in Table 1 stratified on 30-day, 90-day, and 1-year mortality. The mean age was 79 years, and 70% were female. The median length of stay was 10 days and declined from 14 to 8 days from 2000 to 2014 (Figure 2). The number of HF decreased from 8,219 in 2000 to 6,556 in 2014 (Figure 3).

**Table 1.** Baseline characteristics of Danish hip fracture patients in the period 2000–2014. Values are frequency (%) and [95% confidence interval] unless otherwise specified

Characteristic	All patients n = 113,721	30-day mortality n = 10,913 (9.6%)	90-day mortality n = 18,412 (16.2%)	1-year mortality n = 30,429 (26.8%)
Age, mean (SD)	78.9 (11.7)	84.6 (8.9)	84.2 (9.1)	83.3 (9.5)
Women	79,161 (69.6) [69.3–69.9]	6,315 (57.9) [56.9–58.8]	11,367 (61.7) [61.0–62.4]	19,352 (63.6) [63.1–64.1]
Length of stay (IQR), days	10 (6–17)	7 (4–12)	8 (4–16)	10 (5–18)
Fracture type **^a^**				
Collum femoris fracture	60,891 (53.5) [53.3–53.9]	5,309 (8.7) [8.5–8.9]	9,128 (15.0) [14.7–15.3]	15,441 (25.4) [25.0–25.7]
Pertrochanteric fracture	44,541 (39.2) [38.9–39.5]	4,700 (10.6) [10.3–10.8]	7,808 (17.5) [17.2–17.9]	12,627 (28.4) [27.9–28.8]
Subtrochanteric fracture	8,289 (7.3) [7.1–7.4]	904 (10.9) [10.2–11.6]	1,476 (17.8) [17.0–18.6]	2,361 (28.5) [27.5–29.5]
Type of operation				
Open/closed reposition	1,251 (1.1) [1.0–1.2]	112 (9.0) [7.4–10.5]	193 (15.4) [13.4–17.4]	331(26.5) [24.0–28.9]
External fixation	98 (0.1) [0.1–0.1]	7 (7.1) [2.0–12.2]	16 (16.3) [9.0–23.6]	23 (23.5) [15.1–31.9]
Internal fixation	82,549 (72.6) [72.2–72.8]	7,677 (9.3) [9.1–9.5]	13,015 (15.8) [15.5–16.0]	21,865 (26.5) [26.2–26.8]
Hemiarthroplasty	24,896 (21.9) [21.7–22.1]	2,686 (10.8) [10.4–11.2]	4,494 (18.1) [17.6–18.5]	7,121 (28.6) [28.0–29.6]
Arthroplasty	4,331 (3.8) [3.7–3.9]	252 (5.8) [5.1–6.5]	439 (10.1) [9.2–11.0]	765 (17.7) [16.5–18.8]
Other	596 (0.5) [0.5–0.6]	179 (30.0) [26.4–33.7]	255 (42.8) [38.8–46.8]	324 (54.4) [504–58.4]
Comorbidities				
Acute myocardial infarction	4,749 (4.2) [4.1–4.3]	905 (19.1) [17.9–20.2]	1,357 (28.6) [27.3–29.9]	1,977 (41.6) [40.2–43.0]
Heart failure	7,904 (7.0) [6.8–7.1]	1,799 (22.8) [21.8–23.7]	2,634 (33.3) [32.3–34.4]	3,843 (48.6) [47.5–49.7]
Cerebrovascular disease	13,099 (11.5) [11.3–11.7]	1,800 (13.7) [13.2–14.3]	2,915 (22.3) [21.5–23.0]	4,644 (35.5) [34.6–36.3]
Peripheral vascular disease	5,208 (4.6) [4.5–4.7]	734 (14.1) [13.2–15.0]	1,192 (22.9) [21.8–24.0]	1,930 (37.1) [35.8–38.4]
Chronic obstructive pulmonary disease	8,734 (7.7) [7.5–7.8]	1,566 (17.9) [17.1–18.7]	2,449 (28.0) [27.1–29.0]	3,791 (43.4) [42.4–44.4]
Kidney disease	2,051 (1.8) [1.7–1.9]	519 (25.3) [23.4–27.2]	725 (35.4) [33.3–37.4]	1,096 (53.4) [51.3–55.6]
Liver disease	1,540 (1.4) [1.3–1.4]	186 (12.1) [10.5–13.7]	295 (19.2) [17.2–21.1]	524 (34.0) [31.7–36.4]
Diabetes	7,916 (7.0) [6.8–7.1]	1,097 (13.9) [13.1–14.6]	1,729 (21.8) [20.9–22.8]	2,795 (35.3) [34.3–36.4]
Rheumatic disease	2,789 (2.5) [2.4–2.5]	256 (9.2) [8.1–10.3]	478 (17.1) [15.7–18.5]	802 (28.8) [27.1–30.4]
Cancer	7,052 (6.2) [6.1–6.3]	1,106 (15.7) [14.8–16.5]	1,933 (27.4) [26.4–28.5]	3,060 (43.4) [42.2–44.6]
Metastasis	1,459 (1.3) [1.2–1.3]	370 (25.4) [23.1–27.6]	669 (45.9) [43.3–48.4]	1,040 (71.3) [69.0–73.6]
Dementia	9,616 (8.5) [8.3–8.6]	1,927 (20.0) [19.2–20.8]	3,166 (32.9) [32.0–33.9]	4,702 (49.0) [47.9–49.9]
Charlson Comorbidity Index				
0	66,097 (58.1) [57.8–58.4]	3,855 (5.8) [5.7–6.0]	6,878 (10.4) [10.2–10.6]	12,200 (18.5) [18.2–18.8]
1–2	30,113 (26.5) [26.2–26.7]	3,641 (12.1) [11.7–12.5]	6,075 (20.2) [19.7–20.6]	9,888 (32.8) [32.3–33.4]
3–4	11,163 (9.8) [9.5–10.0]	1,948 (17.5) [16.8–18.2]	3,111 (27.9) [27.0–28.7]	4,773 (42.8) [41.8–43.7]
≥ 5	6,348 (5.6) [5.5–5.7]	1,469 (23.1) [22.1–24.2]	2,348 (37.0) [35.8–38.2]	3,568 (56.2) [55.0–57.4]

Abbreviations: 95% CI = 95% confidence interval; IQR = interquartile range; SD = standard deviation

a From here down, the percentages in 30-day, 90-day, and 1-year mortality are calculated by rows.

### Mortality

**Figure 4.**
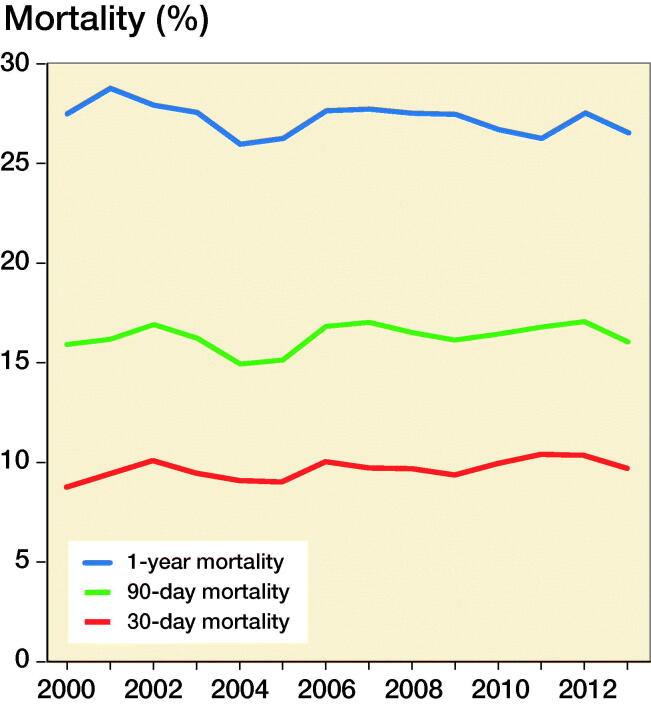
Trends in hip fracture mortality from 2000 to 2013. The year 2014 is missing since follow-up ended in 2014. Logistic regression of periodic effect, 1-year mortality trend, p < 0.001; 90-day mortality trend, p = 0.9; and 30-day mortality trend, p = 0.1.

The postoperative mortality risk was 9.6% for 30-day, 16% for 90-day, and 27% for 1-year. From 2000 to 2013, the postoperative 30-day, 90-day, and 1-year mortality risks showed no clear trend towards an increase or decrease (Figure 4). Cardiovascular death was the most common; at 1 year 27% of patients had died of cardiovascular disease and ischemic heart disease accounted for 17% of the total deaths (Table 2, see Supplementary data).

Subtrochanteric and pertrochanteric fractures were associated with an increased risk of mortality, and patients undergoing a total hip arthroplasty seemed to have a reduced risk of mortality in the uni- and multivariable Cox regression analysis (Tables 3 and 4, see Supplementary data). In both the uni- and multivariable Cox regression male sex, increasing age, and CCI were statistically significantly associated with mortality. We found a statistically significant interaction between sex and age in all analyses. Moreover, the majority of the individual organ-specific comorbidities were statistically significantly associated with an increased risk of postoperative mortality (Table 4). Both existing cerebrovascular and rheumatic disease were associated with a reduced risk of postoperative mortality. 

## Discussion

This study found a 30-day postoperative mortality risk of 9.6%, 90-day of 16%, and 1-year of 27%. Over a 15-year period (2000–2014), the mortality risk varied but with no overall increase or decrease over time. Male sex, increasing age, higher CCI score, type of fracture, and operation were independently associated with postoperative mortality. Comorbidities such as kidney and liver disease, COPD, heart failure, dementia, and cancer with metastases had a strong association with short- and long-term postoperative mortality while the presence of a cerebrovascular or rheumatic disease decreased the mortality risk. Length of stay decreased from 2000 to 2014, from a median of 14 to 8 days. The number of surgical interventions for first-time HF was also found to have decreased in the period 2000 to 2014.

Surprisingly, we found that patients with a known cerebrovascular disease had a decreased risk of postoperative mortality. These patients are treated with preventive anticoagulant drugs, thus the finding might reflect the preventive effect of anticoagulant medication on postoperative cardiovascular events and mortality. Regarding the decreased risk of postoperative mortality in patients with known rheumatoid arthritis, the improvements in the treatment of rheumatoid arthritis in the past decades could potentially explain the study result. No conclusion can be drawn on these matters, which should be further investigated.

In the uni- and multivariate Cox regression, we found an association between type of fracture, type of operation, and the risk of postoperative mortality. There exists no international consensus on whether the type of fracture influences postoperative mortality following HF surgery. One study with 41,000 patients found that patients with a collum femoris fracture had a longer mean survival time compared with patients with a trochanteric fracture (Kannegaard et al. 2010) whereas a smaller study with 428 patients found no association (Panula et al. 2011). A study from the American College of Surgeons’ National Surgical Quality Improvement database including around 9,500 patients with hip fractures found no difference in mortality comparing different types of operations (Sathiyakumar et al. 2015). However, a retrospective register study with 11,253 Swedish patients found that total hip arthroplasty had a lower mortality rate compared with hemiarthroplasty (Hansson et al. 2017). Further studies are needed to shed light on whether the type of operative procedure influences the postoperative mortality following HF surgery and whether the prognosis of specific comorbid elderly patients can be modulated by choosing procedures with less trauma and thus less surgical stress. Even though the multivariable analysis was adjusted for age and background comorbidities, these results may reflect confounding by indication and should be interpreted with caution. Patients may have been pre-selected for certain surgical interventions based on their pre-surgical characteristics.

In recent years, there has been a high awareness of fast-track surgery. The goal is to operate on the patient within 24 hours of admission, achieve fast postoperative mobilization, adequate nutrition, and minimize the use of analgesic drugs (Kehlet and Dahl 2003). The time from admission with an HF to surgery correlates to increased mortality (Liu et al. 2017), and with fast-track surgery a decrease in the postoperatively mortality has been reported (Pedersen et al. 2008). We found a decline in median length of stay from 14 in 2000 to 8 days in 2014, but on a national level we found postoperative mortality to be relatively unchanged. National implementation of fast-track HF surgery could potentially improve the postoperative course (Egerod et al. 2010).

This study has limitations as it is an observational study and unmeasured confounding is present. The patients were selected by diagnosis and operation codes in the DNPR. Therefore, patients with incorrect coding may not be included; however, the DNPR is validated (Lynge et al. 2011). We did not have data on patients’ daily medicine consumption, smoking, and alcohol intake as well as the patient’s physiological status including BMI, disease severity, frailty, and sociodemographic status. Furthermore, the mortality of patients with HF was not compared with a normal population, and it is therefore not clear whether the mortality is higher in this group. Postoperative complication is not part of this study design but could be relevant in future studies.

In summary, in this national Danish cohort study, patients undergoing surgery for a hip fracture had postoperative mortality of 9.6% at 30 days, 16% at 90 days and 27% at 1 year. The postoperative mortality varied between years but did not decline from 2000 to 2013. But the length of stay declined from a median of 14 days in 2000 to 8 days in 2014. Further interventional studies must be undertaken in order to improve survival after HF surgery, and potentially study whether a national fast-track program could improve postoperative survival, as seen in regional studies.  

### Supplementary data

Tables 2–4 are available as supplementary data in the online version of this article, http://dx.doi.org/10.1080/17453674.2019.1680485

## Supplementary Material

Supplemental Material
