# Supplementary material for: Postoperative mortality after a hip fracture over a 15-year period in Denmark: a national register study
Source: Acta Orthop. 2019 Oct 22;91(1):58–62. doi: 10.1080/17453674.2019.1680485 (PMC7006693; doi:10.1080/17453674.2019.1680485)
Supplement: Supplemental Material [file IORT_A_1680485_SM3002.pdf]

Supplementary data

Table 2. Cause of death in patients with HF from 2000 to 2014 distributed among 30-day, 90-day, and 1-year mortality. Values are frequency (%) and [95% confidence interval]

Cause of death	30-day mortality n = 10,913		90-day mortality n = 18,412		1-year mortality n = 30,429	
Accidents and lesions	4,552 (41.7)	[40.8–42.6]	5,712 (31.0)	[30.3–31.7]	6,278 (20.6)	[20.2–21.1]
Cardiovascular disease	2,386 (21.9)	[21.9–22.6]	4,340 (23.6)	[23.0–24.2]	8,075 (26.5)	[26.0–27.0]
Cancer	978 (9.0)	[8.4–9.5]	2,255 (12.3)	[11.8–12.7]	4,618 (15.2)	[14.8–15.6]
Respiratory disease	977 (9.0)	[8.4–9.5]	1,774 (9.6)	[9.2–10.1]	3,207 (10.5)	[10.2–10.9]
Mental illness	435 (4.0)	[3.6–4.4]	1,022 (5.6)	[5.2–5.9]	1,928 (6.3)	[6.1–6.6]
Unspecific	455 (4.2)	[3.8–4.5]	944 (5.1)	[4.8–5.5]	1,783 (5.9)	[5.6–6.1]
Gastrointestinal disease	387 (3.6)	[3.2–3.9]	751 (4.1)	[3.8–4.4]	1,368 (4.5)	[4.3–4.7]
Neurological disease	199 (1.8)	[1.6–2.1]	446 (2.4)	[2.2–2.6]	891 (2.9)	[2.7–3.1]
Endocrinological disease	183 (1.7)	[1.4–1.9]	393 (2.1)	[1.9–2.3]	830 (2.7)	[0.5–2.9]
Infections	111 (1.0)	[0.8–1.2]	280 (1.5)	[1.3–1.7]	522 (1.7)	[1.6–1.9]
Urogenital disease	128 (1.2)	[1.0–1.4]	248 (1.4)	[1.2–1.5]	462 (1.5)	[1.4–1.7]
Connective tissue disorder	77 (0.7)	[0.6–0.9]	144 (0.8)	[0.7–0.9]	261 (0.9)	[0.8–1.0]
Immune system disease	33 (0.3)	[0.2–0.5]	65 (0.4)	[0.3–0.4]	129 (0.4)	[0.4–0.5]
Skin disease	8 (0.1)	[0.0–0.1]	26 (0.1)	[0.1–0.2]	57 (0.2)	[0.1–0.2]
Congenital disease	< 5 (< 0.1)	[0.0–0.0]	11 (0.1)	[0.0–0.1]	18 (0.1)	[0.0–0.1]
Eye disease	0 (0.0)	[0.0–0.0]	< 5 (< 0.1)	[0.0–0.0]	< 5 (< 0.1)	[0.0–0,0]

Table 3. Univariable Cox regression model of 30-day, 90-day, and 1-year postoperative mortality of Danish hip fracture patients in the period 2000–2014

Covariates	30-day mortality HR (95% CI)	p-value	90-day mortality HR (95% CI)	p-value	1-year mortality HR (95% CI)	p-value
Sex						
Female	1.00 (REF)	< 0.001	1.00 (REF)	< 0.001	1.00 (REF)	< 0.001
Male	1.94 (1.87–2.02)		1.70 (1.65–1.75)		1.60 (1.55–1.63)	
Age, years						
< 70	1.00 (REF)	< 0.001	1.00 (REF)	< 0.001	1.00 (REF)	< 0.001
70–79	1.95 (1.78–2.13)		1.83 (1.71–1.96)		1.72 (1.64–1.80)	
80–89	3.55 (3.27–3.86)		3.27 (3.08–3.48)		2.90 (2.77–3.03)	
≥ 90	7.20 (6.60–7.84)		6.49 (6.09–6.91)		5.35 (5.10–5.60)	
Fracture type						
Collum femoris fracture	1.00 (REF)	< 0.001	1.00 (REF)	< 0.001	1.00 (REF)	< 0.001
Pertrochanteric fracture	1.24 (1.19–1.31)		1.18 (1.13–1.22)		1.07 (1.04–1.10)	
Subtrochanteric fracture	1.45 (1.34–1.56)		1.34 (1.26–1.42)		1.20 (1.15–1.26)	
Type of operation						
Closed reposition	1.00 (REF)	< 0.001	1.00 (REF)	< 0.001	1.00 (REF)	< 0.001
Open reposition	1.05 (0.68–1.62)		0.95 (0.67–1.34)		1.08 (0.84–1.39)	
External fixation	0.90 (0.42–1.94)		1.12 (0.67–1.88)		0.91 (0.59–1.40)	
Internal fixation	1.01 (0.82–1.26)		0.98 (0.83–1.15)		0.99 (0.87–1.12)	
Hemiarthroplasty	1.24 (1.00–1.54)		1.13 (0.96–1.33)		1.02 (0.90–1.16)	
Arthroplasty	0.90 (0.70–1.15)		0.83 (0.69–1.00)		0.77 (0.67–0.89)	
Other	3.34 (2.57–4.32)		2.88 (2.35–3.52)		2.33 (1.98–2.75)	
Comorbidities [a]						
Acute myocardial infarct	1.11 (1.02–1.22)	0.02	1.08 (1.01–1.16)	0.04	1.00 (0.93–1.05)	0.8
Heart failure	1.42 (1.31–1.53)	< 0.001	1.32 (1.24–1.41)	< 0.001	1.24 (1.18–1.31)	< 0.001
Cerebrovascular disease	0.91 (0.86–0.98)	0.01	0.91 (0.85–0.97)	0.003	0.90 (0.85–0.94)	< 0.001
Peripheral vascular disease	0.95 (0.86–1.04)	0.3	0.98 (0.91–1.05)	0.5	0.98 (0.92–1.04)	0.4
Chronic obstructive pulmonary disease	1.40 (1.29–1.51)	< 0.001	1.37 (1.29–1.46)	< 0.001	1.33 (1.26–1.40)	< 0.001
Kidney disease	1.82 (1.66–1.98)	< 0.001	1.69 (1.56–1.82)	< 0.001	1.72 (1.61–1.83)	< 0.001
Liver disease	1.50 (1.23–1.70)	< 0.001	1.37 (1.20–1.56)	< 0.001	1.37 (1.24–1.51)	< 0.001
Diabetes	1.15 (1.07–1.23)	< 0.001	1.12 (1.06–1.18)	< 0.001	1.11 (1.06–1.16)	< 0.001
Rheumatic disease	0.75 (0.66–0.86)	< 0.001	0.83 (0.75–0.92)	< 0.001	0.82 (0.76–0.89)	< 0.001
Cancer	1.19 (1.09–1.30)	< 0.001	1.33 (1.24–1.42)	< 0.001	1.33 (1.26–1.40)	< 0.001
Cancer metastasis	2.24 (1.83–2.74)	< 0.001	2.70 (2.30–3.16)	< 0.001	2.73 (2.40–3.10)	< 0.001
Dementia	1.40 (1.29–1.51)	< 0.001	1.47 (1.38–1.56)	< 0.001	1.40 (1.33–1.48)	< 0.001
Charlson Comorbidity Index						
0	1.00 (REF)	< 0.001	1.00 (REF)	< 0.0001	1.00 (REF)	< 0.001
1–2	1.67 (1.55–1.81)		1.61 (1.51–1.71)		1.60 (1.52–1.68)	
3–4	2.08 (1.82–2.38)		1.98 (1.78–2.20)		1.98 (1.82–2.16)	
≥ 5	2.37 (1.91–2.93)		2.26 (1.91–2.67)		2.37 (2.06–2.71)	

[a] The comorbidity hazard ratio where found by comparing with non-comorbid patients.

Table 4. Multivariable Cox regression model of 30-day, 90-day, and 1-year postoperative mortality in the period 2000–2014

Covariates	30-day mortality HR (95% CI)	p-value	90-day mortality HR (95% CI)	p-value	1-year mortality HR (95% CI)	p-value
Age, years						
Risk estimates of age for female patients						
< 70	1.00 (REF)	< 0.001	1.00 (REF)	< 0.001	1.00 (REF)	< 0.001
70–79	1.44 (1.27–1.64)		1.50 (1.37–1.65)		1.46 (1.37–1.56)	
80–89	2.55 (2.27–2.87)		2.59 (2.37–2.82)		2.41 (2.27–2.56)	
≥ 90	5.28 (4.69–5.94)		5.22 (4.80–5.70)		4.49 (4.22–4.79)	
Risk estimates of age for male patients						
< 70	1.16 (1.00–1.35)		1.17 (1.05–1.31)		1.18 (1.09–1.28)	
70–79	2.79 (2.46–3.17)		2.49 (2.26–2.74)		2.31 (2.16–2.48)	
80–89	5.28 (4.70–5.94		4.67 (4.28–5.09		4.02 (3.77–4.28)	
≥ 90	10.44 (9.21–11.84)		9.02 (8.20–9.92)		7.36 (6.85–7.91)	
Fracture type						
Collum femoris fracture	1.00 (REF)	< 0.001	1.00 (REF)	< 0.001	1.00 (REF)	< 0.001
Pertrochanteric fracture	1.25 (1.19–1.31)		1.18 (1.14–1.23)		1.07 (1.04–1.11)	
Subtrochanteric fracture	1.46 (1.35–1.57)		1.35 (1.27–1.43)		1.21 (1.15–1.26)	
Type of operation						
Closed reposition	1.00 (REF)	< 0.001	1.00 (REF)	< 0.001	1.00 (REF)	< 0.001
Open reposition	1.06 (0.68–1.63)		0.96 (0.68–1.34)		1.09 (0.84–1.39)	
External fixation	0.91 (0.42–1.96)		1.13 (068–1.90)		0.92 (0.60–1.41)	
Internal fixation	1.01 (0.82–1.26)		0.98 (0.83–1.15)		0.99 (0.87–1.12)	
Hemiarthroplasty	1.24 (1.00–1.55)		1.13 (0.96–1.34)		1.02 (0.90–1.16)	
Arthroplasty	0.90 (0.70–1.15)		0.83 (0.69–1.00)		0.77 (0.67–0.89)	
Other	3.34 (2.58–4.33)		2.88 (2.35–3.53)		2.34 (1.98–2.76)	
Comorbidities [a]						
Acute myocardial infarct	1.11 (1.01–1.22)	0.03	1.08 (1.00–1.16)	0.05	0.99 (0.93–1.05)	0.7
Heart failure	1.41 (1.30–1.53)	< 0.001	1.31 (1.23–1.40)	< 0.001	1.24 (1.17–1.30)	< 0.001
Cerebrovascular disease	0.90 (0.84–1.94)	0.01	0.91 (0.85–0.97)	0.002	0.89 (0.85–0.94)	< 0.001
Peripheral vascular disease	0.94 (0.86–1.04)	0.2	0.97 (0.90–1.05)	0.5	0.97 (0.92–1.03)	0.4
Chronic obstructive pulmonary disease	1.39 (1.28–1.50)	< 0.001	1.36 (1.28–1.45)	< 0.001	1.32 (1.26–1.39)	< 0.001
Kidney disease	1.81 (1.65–1.98)	< 0.001	1.68 (1.55–1.81)	< 0.001	1.71 (1.61–1.82)	< 0.001
Liver disease	1.48 (1.26–1.74)	< 0.001	1.39 (1.22–1.59)	< 0.001	1.39 (1.26–1.54)	< 0.001
Diabetes	1.15 (1.07–1.23)	< 0.001	1.12 (1.06–1.18)	< 0.001	1.11 (1.06–1.16)	< 0.001
Rheumatic disease	0.75 (0.65–0.86)	< 0.001	0.83 (0.75–0.92)	< 0.001	0.82 (0.76–0.89)	< 0.001
Cancer	1.18 (1.08–1.29)	< 0.001	1.32 (1.23–1.41)	< 0.001	1.32 (1.25–1.39)	< 0.001
Cancer metastasis	2.20 (1.80–2.69)	< 0.001	2.66 (2.27–3.11)	< 0.001	2.69 (2.36–3.06)	< 0.001
Dementia	1.39 (1.29–1.51)	< 0.001	1.46 (1.38–1.56)	< 0.001	1.40 (1.33–1.47)	< 0.001
Charlson Comorbidity Index						
0	1.00 (REF)	< 0.001	1.00 (REF)	< 0.001	1.00 (REF)	< 0.001
1–2	1.68 (1.55–1.81)		1.61 (1.51–1.71)		1.60 (1.52–1.68)	
3–4	2.09 (1.83–2.39)		1.99 (1.79–2.22)		1.99 (1.83–2.17)	
≥ 5	2.39 (1.93–2.96)		2.27 (1.92–2.69)		2.38 (2.08–2.73)	

[a] The comorbidity hazard ratio where found by comparing with non-comorbid patients.
